# Machine Learning and Deep Learning Approaches in Lifespan Brain Age Prediction: A Comprehensive Review

**DOI:** 10.3390/tomography10080093

**Published:** 2024-08-12

**Authors:** Yutong Wu, Hongjian Gao, Chen Zhang, Xiangge Ma, Xinyu Zhu, Shuicai Wu, Lan Lin

**Affiliations:** Department of Biomedical Engineering, College of Chemistry and Life Science, Beijing University of Technology, Beijing 100124, China; wyt191026@emails.bjut.edu.cn (Y.W.); gaohongjian@bjut.edu.cn (H.G.); zc202265180@emails.bjut.edu.cn (C.Z.); maxiangge@emails.bjut.edu.cn (X.M.); zhuxyu@emails.bjut.edu.cn (X.Z.); wushuicai@bjut.edu.cn (S.W.)

**Keywords:** machine learning, deep learning, brain age prediction, lifespan brain age, neuroimaging

## Abstract

The concept of ‘brain age’, derived from neuroimaging data, serves as a crucial biomarker reflecting cognitive vitality and neurodegenerative trajectories. In the past decade, machine learning (ML) and deep learning (DL) integration has transformed the field, providing advanced models for brain age estimation. However, achieving precise brain age prediction across all ages remains a significant analytical challenge. This comprehensive review scrutinizes advancements in ML- and DL-based brain age prediction, analyzing 52 peer-reviewed studies from 2020 to 2024. It assesses various model architectures, highlighting their effectiveness and nuances in lifespan brain age studies. By comparing ML and DL, strengths in forecasting and methodological limitations are revealed. Finally, key findings from the reviewed articles are summarized and a number of major issues related to ML/DL-based lifespan brain age prediction are discussed. Through this study, we aim at the synthesis of the current state of brain age prediction, emphasizing both advancements and persistent challenges, guiding future research, technological advancements, and improving early intervention strategies for neurodegenerative diseases.

## 1. Introduction

Human development and aging have traditionally been assessed through various physiological and physical changes over an individual’s lifespan [1,2]. These changes encompass a wide range of biological processes, such as alterations in muscle mass, bone density, and cognitive function. However, there is substantial individual variation in how these processes unfold, influenced by a complex interplay of factors, including lifestyle, living environment, and genotype [3,4]. Consequently, individuals with the same chronological age (CA) may exhibit different physiological functions and capabilities, with notable differences even observed among twins [5,6]. Therefore, relying solely on traditional CA often proves inadequate in accurately reflecting an individual’s true biological state. To overcome this limitation, the concept of biological age (BA) has been introduced. BA aims to provide a more precise reflection of an individual’s physiological condition and aging process by integrating various biomarkers and health indicators, offering a more nuanced understanding than CA alone [7]. Recent advances in neuroimaging and machine learning (ML) have significantly enhanced our ability to assess biological age, particularly in the context of brain aging. Neuroimaging techniques, when coupled with ML algorithms, can analyze vast amounts of imaging data to identify patterns and biomarkers indicative of an individual’s brain age. These data-driven approaches have enabled researchers to accurately estimate individual brain age, providing deeper insights into the aging process and enabling the detection of deviations from the expected aging trajectory [8,9,10,11]. Consequently, the concept of brain age gap (BAG) has emerged as a critical metric in this domain. BAG represents the difference between an individual’s predicted brain age, as determined by neuroimaging and ML models, and their CA. A positive BAG indicates that the brain appears older than the individual’s CA, which may be associated with various neurodegenerative conditions or accelerated aging processes. Conversely, a negative BAG suggests that the brain is younger than expected, potentially reflecting better cognitive health and resilience against age-related decline [12,13].

Predicting brain age across all age groups—from infants and young adults to middle-aged and elderly individuals—holds substantial significance in both neuroscience and clinical practice. For infants and young children, early brain age estimation is pivotal in identifying developmental delays or neurological disorders, facilitating timely interventions and support during crucial developmental periods [14]. In young adults, understanding brain age can aid in recognizing early signs of mental health conditions or cognitive impairments that may not yet manifest behaviorally, enabling preemptive strategies to maintain cognitive health [15]. As individuals transition into middle age, brain age predictions become essential for monitoring cognitive health, particularly considering lifestyle factors and comorbidities that influence brain aging [16,17]. This age group can benefit from tailored interventions aimed at mitigating cognitive decline and promoting neuroplasticity. Furthermore, in elderly populations, brain age estimation is critical for the early detection of neurodegenerative diseases, such as Alzheimer’s and Parkinson’s [18,19]. It allows more accurate prognostic evaluations and the implementation of therapeutic measures to slow disease progression and improve quality of life. Understanding the brain age across the full spectrum of life stages can contribute to the broader scientific understanding of the aging process itself. It can help researchers identify patterns and anomalies in cognitive aging, leading to the development of targeted therapies and lifestyle interventions that promote brain health and resilience against age-related cognitive decline.

A series of review articles have elucidated methods for predicting brain age using neuroimaging data. These reviews primarily discuss the application of ML methods in brain age prediction, thereby enhancing our understanding of neuroimaging data-driven tools for this purpose. For example, Jirsaraie et al. conducted a systematic review of multimodal brain age studies in 2023 to identify the most significant neuroimaging features [20]. Tanveer et al. provided an extensive discussion on research in the brain age prediction field from 2017 to March 2022, offering a comprehensive evaluation of the deep learning (DL) models that have substantially contributed to the existing body of knowledge [2]. Kumari et al. examined various techniques and potential therapeutic applications of brain age prediction [21], while Seitz-Holland et al. systematically reviewed brain age findings in neuropsychiatric disorders, highlighting the potential of brain age as a biomarker for biological aging [22]. Predicting brain age across the entire age spectrum is a crucial yet challenging endeavor in this field. Models that encompass the full range of human development and aging offer superior comparability and robustness compared to those focused on specific age groups. However, accurately predicting brain age throughout the entire lifespan necessitates sophisticated model design to accommodate the complex and varied changes occurring at different life stages. Despite the significance of this task, current reviews in the field tend to provide broad overviews, lacking in-depth analysis of this critical area. To address this gap, we conducted a thorough review of the advancements in ML and DL for lifespan brain age prediction from 2020 to 2024, encompassing peer-reviewed studies. This review aims to provide a more detailed and comprehensive understanding of the latest developments and challenges in predicting brain age across the lifespan. By comparing ML and DL methods, we reveal their predictive advantages and methodological limitations. We not only elucidate these recent advancements and discoveries but also carefully discuss the main issues in ML- and DL-based brain age prediction. Through this study, we aim to clarify the current state of brain age prediction, highlight the progress and ongoing challenges, and guide future research.

## 2. Literature Search Strategy

**Figure 1:**
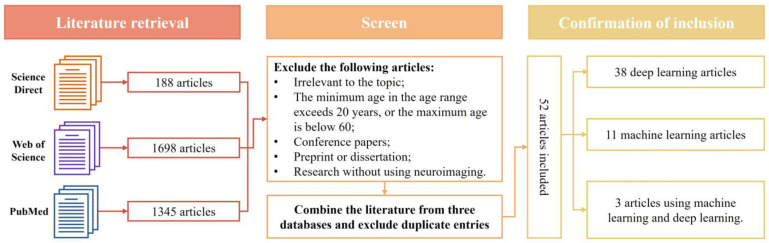
A schematic diagram of the literature search strategy.

We conducted a comprehensive search of three commonly used literature databases to identify relevant studies: PubMed, ScienceDirect, and Web of Science. The search was confined to articles published between 2020 and 2024, using the following keywords: (((machine learning) OR (deep learning) OR (CNN) OR (Transformer)) AND ((brain age) OR (brain biological age)) AND ((prediction) OR (estimation))). Detailed inclusion and exclusion criteria for selecting articles included in this study are provided in Figure 1.

Initially, the search yielded 3231 relevant research papers. To ensure maximum relevance, we performed an iterative selection process. Papers were first excluded if they were deemed unrelated to the topic or had age range criteria that did not meet our thresholds (minimum age above 20 or maximum age below 60). Additionally, conference records, preprint manuscripts, theses, and studies that did not use neuroimaging were excluded during this phase. Duplicate articles from the literature databases were subsequently removed. Following a thorough and meticulous evaluation of all the remaining articles, a total of 52 studies were included in the final review, as they clearly met the inclusion criteria for this review.

## 3. Fundamental of Brain Age Predictions Tasks

### 3.1. Neuroimage Data and Preprocessing Process

In the task of brain age prediction, neuroimaging data play a fundamental role, providing valuable insights into the complex changes associated with age-related brain development and aging processes. Different data modalities contribute to a comprehensive understanding of the brain’s aging process. Commonly used modalities include T1 magnetic resonance imaging (MRI), T2 MRI, diffusion tensor imaging (DTI), and function MRI (fMRI). It is noteworthy that T1 MRI and T2 MRI data offer structural insights into the brain, serving as a valuable window for detecting irregularities in gray matter (GM) and white matter (WM). These modalities excel in describing age-related developmental and atrophic changes, as well as in identifying morphological alterations such as white matter lesions. In contrast, DTI is an indispensable tool for studying age-related changes in white matter connectivity. By examining the diffusion of water molecules within neural fibers, DTI provides a detailed investigation of the microstructure of white matter. Additionally, fMRI is crucial for uncovering functional changes in the brain, as it can detect blood flow and neural activity. This makes it an important tool for exploring patterns of functional connectivity and revealing the underlying dynamics of brain function.

When incorporating neuroimaging data, such as T1-weighted MRI, into DL or ML workflows, rigorous preprocessing is an essential step. This process ensures that the data are standardized and optimized for analysis, minimizing potential biases or errors. Toolkits such as Statistical Parametric Mapping (SPM, https://www.fil.ion.ucl.ac.uk/spm/ (accessed on 7 August 2024)), FreeSurfer (https://surfer.nmr.mgh.harvard.edu/ (accessed on 7 August 2024)), and FMRIB Software Library (FSL, https://fsl.fmrib.ox.ac.uk/fsl/docs/#/ (accessed on 7 August 2024)) facilitate this process. Typically, T1 MRI data are segmented into gray matter (GM), white matter (WM), and cerebrospinal fluid (CSF), with these segments fed into DL models as 3D density maps or 2D slices. In ML studies, detailed segmentation using brain atlases, such as the AAL atlas, allows the extraction of features like cortical thickness and volume. The use of advanced methods, such as the teacher–student model can further enhance data quality [23].

### 3.2. ML and DL Models

In the realm of brain age prediction, the employment of ML and DL models has revolutionized the approach towards understanding the intricate processes of brain development and aging.

ML, a subset of artificial intelligence, has undergone a significant evolution, presenting a wide array of algorithms tailored for extracting insights from neuroimaging data and continuously enhancing performance. Key to its application in neuroimaging studies is the ability to discern intricate patterns that correlate brain images with age-related metrics. These patterns are unveiled through a spectrum of supervised learning techniques, each meticulously designed to extract knowledge from annotated datasets.

**Figure 2:**
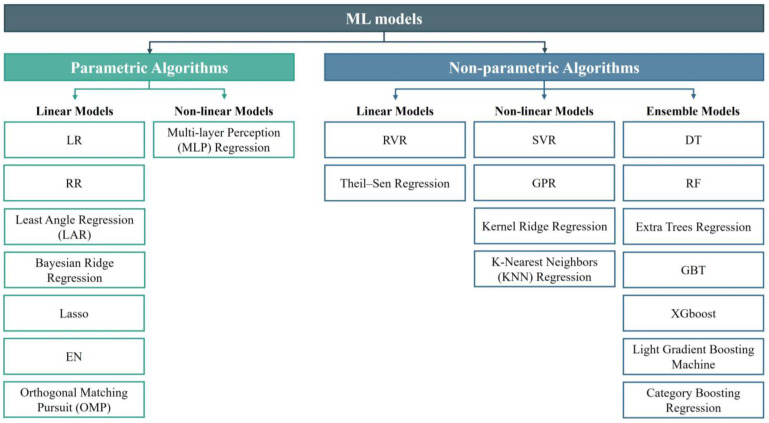
Types of ML algorithms.

Figure 2 illustrates the types of ML models. ML models can be categorized into parametric and non-parametric models based on their structure and learning approach [24]. Parametric models are those that can be defined by a finite set of parameters. The primary advantage of these models is the significant simplification of the learning process, while also constraining the scope of learning. Linear regression (LR) is a fundamental ML method that fits a linear model by minimizing the sum of squared residuals between observed values and the predictions. Extensions of LR, such as ridge regression (RR), Lasso regression, and elastic net regression (EN), incorporate regularization techniques to address potential overfitting through penalty terms on coefficient magnitudes. However, due to the parametric models’ prior assumptions about data distribution, they struggle to handle complex data patterns and tend to exhibit significant bias when the training data are insufficient. Models that do not make extensive assumptions about the form of the target function are called non-parametric models. These include decision trees (DTs), support vector regression (SVR), neural networks, Gaussian process regression (GPR), and others. Non-parametric models are more flexible and capable of capturing complex patterns and structures in the data. SVR excels in both linear and nonlinear classification and regression tasks by identifying optimal hyperplanes within high-dimensional spaces. In the realm of neuroimaging, SVR excels in handling complex, multidimensional data and detecting nuanced patterns associated with the aging brain. Relevance vector regression (RVR), a sparse regression model based on Bayesian theory, utilizes Bayesian inference to determine which training samples are relevance vectors, ultimately generating a sparse model [10]. Compared to SVR, RVR does not require explicit setting of regularization parameters and epsilon. Instead, it automatically determines the relevance vectors through the Bayesian inference process. GPR distinguishes itself through its probabilistic approach, offering predictive distributions rather than singular estimates [25]. This capability strengthens GPR’s capacity to quantify uncertainty in brain age predictions, which is crucial for making informed clinical decisions. Random forests (RFs), composed of ensembles of DT, bolster robustness by aggregating predictions from multiple weaker learners [26]. They effectively mitigate the risks of overfitting and enhance model generalizability, establishing their reliability in predicting brain age based on imaging features. Gradient boosting trees (GBTs) methodically construct DT ensembles to iteratively minimize loss functions, adeptly capturing the intricate data relationships essential for precise estimation [27]. Additionally, extreme gradient boosting (XGBoost) stands out for its efficiency in handling vast datasets and delivering precise predictions, leveraging its regularization properties to optimize performance [28]. The suite of algorithms presented, spanning from traditional regression methodologies to sophisticated ensemble and tree-based models, represents a collaborative effort aimed at advancing the frontier of brain age prediction capabilities.

**Figure 3:**
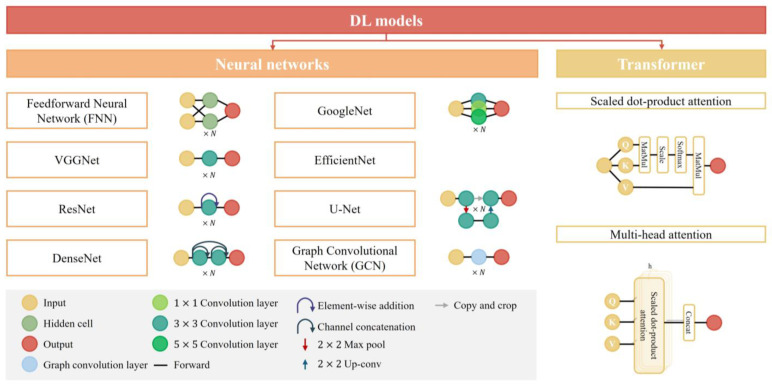
Types of DL algorithms. The model architecture shown in the figure is not the complete structure, the full architecture typically consists of *N* similar modules. In the figure of the Transformer architecture, Q, K, and V represent “Query”, “Key”, and “Value”, respectively, while h denotes the number of attention heads.

DL, a specialized field within ML, involves algorithms inspired by the structure and function of the brain’s neural networks. It operates through artificial neural networks comprising layers of interconnected nodes, or neurons, that process information hierarchically. This architecture, particularly convolutional neural networks (CNNs), enables deep learning models to automatically learn representations of data, often leading to superior performance in image-based tasks [29]. CNNs are composed of several modules, each serving distinct functions and effectively combined to achieve feature extraction from neuroimages. The convolutional layers, aptly named, perform convolution operations to extract image features. Pooling layers then perform parameterized subsampling on feature maps to reduce image dimensions. Nonlinear activation functions map input data to corresponding outputs, enabling neural networks to model complex relationships in the data. Fully connected layers, commonly used as the final layer in CNN architectures, utilize these extracted features for regression and classification tasks. The loss function plays a crucial role in guiding weight adjustments within the CNN architecture. Significant advancements have been made in developing efficient network architectures, exemplified by VGGNet, ResNet, GoogleNet, DenseNet, EfficientNet, and others. Figure 3 illustrates the types of DL models. These architectures have demonstrated outstanding performance in brain age prediction tasks across the entire age spectrum, underscoring the importance of thoughtful architectural design in leveraging CNNs for effective feature extraction and modeling complex relationships in neuroimaging data.

The transformative impact of DL is further amplified by advanced models such as Transformers. Through self-attention mechanisms, Transformers can capture long-range dependencies in the data, which is particularly relevant in understanding the progression of brain aging. This concept is often extended to multi-head attention mechanisms, where each head can learn different attention weights to capture various types of relationships more effectively [30]. Originally designed for natural language processing (NLP) and sequence-to-sequence tasks, Transformers have been adapted for brain age prediction tasks across the entire age spectrum. By integrating Transformers with CNNs, researchers have explored rich network architecture paradigms, further enhancing the performance and applicability of DL in this domain.

### 3.3. The Evaluation Metric for Brain Age Prediction Tasks

The accuracy of brain age prediction models is typically assessed by calculating the mean absolute error (MAE) across all subjects in the test set, which indicates the difference between the predicted age and the CA. The formula for *MAE* is:

$$MAE=\frac{1}{n}\sum_{i=1}^{n}\left|\hat{y_{i}}-y_{i}\right|$$

where n represents the number of subjects in the test set, $\hat{y_{i}}$ denotes the predicted brain age for the i-th subject, and $y_{i}$ denotes the CA of the i-th subject. However, a single metric may not provide a comprehensive evaluation of the model’s performance, as *MAE* can be misleading, especially when the age range of the test set subjects is overrepresented in the training set. The additional use of the correlation coefficient (*R-values*) as an evaluation metric can help mitigate this issue. *R-values* reflect the goodness of fit of the predicted age to the CA, thereby assessing the model’s performance. The formula for *R-values* is:

$$R-values=\frac{\sum_{i=1}^{n}\left(y_{i}-\bar{Y}\right)\left(\hat{y_{i}}-\bar{\hat{Y}}\right)}{\sqrt{\sum_{i=1}^{n}{\left(y_{i}-\bar{y_{i}}\right)}^{2}}\sqrt{\sum_{i=1}^{n}{\left(\hat{y_{i}}-\bar{\hat{y_{i}}}\right)}^{2}}}$$

where $\bar{Y}$ represents the mean CA in the test set, and $\bar{\hat{Y}}$ denotes the mean predicted brain age in the test set.

## 4. A Review of Brain Age Prediction Model

### 4.1. Brain Age Prediction Using ML Methods

Table 1 provides a summary of the datasets, age ranges, number of subjects, and model performance metrics for brain age prediction tasks using ML algorithms across the entire age spectrum. Researchers primarily focus on optimizing existing ML algorithms and models, as well as on sophisticated feature engineering techniques to enhance predictive accuracy.

#### 4.1.1. Single-Modality Model

T1 MRI data are the most used modality for brain age prediction tasks spanning the entire age spectrum. Researchers have explored various feature extraction methods and ML models to obtain more accurate predictions. For example, Tesli et al. used preprocessed T1 MRI data and utilized FreeSurfer to extract 34 cortical thickness and area region of interest (ROI) values, as well as the average thickness, total area, etc. They employed RF and XGBoost methods for brain age prediction [33]. Similarly, Han et al. [38] and Lee et al. [39] employed the same tools and atlases as Tesli et al. to generate measurements of total intracranial volume, cortical thickness, surface area, and subcortical volumes as input features for their models. Their studies indicated that different types of ML methods exhibited similar predictive performances. Ballester et al. conducted a study using an XGBoost model, utilizing volume, area, and thickness measures as model input features [34]. More et al. designed a comprehensive comparative experiment involving 16 feature representations and 8 different ML methods to explore the optimal workflow. The results indicated that the workflow ‘smoothed with a 4 mm FWHM kernel and resampled to 4 mm spatial resolution + GPR’ performed the best [36]. Similarly, a study by Kalc et al. using the GPR method trained and processed eight single-tissue-type models (GM/WM combinations) with different spatial resolutions and FWHM Gaussian kernel smoothing parameters in the same manner, and PCA was used for data regularization [40]. Additionally, Ly et al. applied mean centering to whole-brain GM density data, followed by the calculation of the dot product, to obtain a similarity matrix as input features for the GPR model [37].

fMRI data have also been applied to brain age prediction. Han et al. utilized the temporal nature of fMRI data, extracting a time series of corresponding brain regions, and they constructed brain functional networks as input features for the different ML models [1].

#### 4.1.2. Multimodality Model

Multimodal data provide ML algorithms with more comprehensive information, but extracting and integrating features from various modalities is challenging. Beck et al. used diffusion MRI (dMRI) data, estimating six modalities: DTI, diffusion kurtosis imaging (DKI), neurite orientation dispersion and density imaging (NODDI), restriction spectrum imaging (RSI), spherical mean technique multi-compartment (SMT mc), and white matter tract integrity (WMTI). They selected 20 scalar metrics and extracted the mean skeleton and 20 ROIs based on a probabilistic white matter atlas. These features were used for brain age prediction with DTI showing the highest accuracy among the single XGBoost models, while a multimodal DT-stacked XGBoost model performed best overall [31]. Similarly, Engemann et al. combined MEG, fMRI, and T1 MRI data. They extracted several temporal and source space features from MEG, ROI-based time series from fMRI, and cortical thickness, surface area, and subcortical volumes from T1 MRI. Using a RR model and an RF general function approximator, they integrated these modalities effectively [32]. Xifra-Porxas et al. fused MEG and T1 MRI data using an RF-stacked GPR model. T1 MRI data were segmented into GM, WM, and CSF, with GM images further subdivided into cortical and subcortical regions. The resulting images were vectorized and then z-scored to obtain feature vectors for each subject. MEG features included power spectral density, amplitude envelope correlation, and cross-frequency coupling. Canonical correlation analysis (CCA) was applied for dimensionality reduction before inputting features, achieving excellent performance [35].

### 4.2. Brain Age Prediction Using DL Methods

Table 2 summarizes the datasets, age ranges, number of subjects, and model performance for the brain age prediction tasks across the entire age spectrum using DL algorithms. Compared to the ML models, DL models offer greater potential for optimization and improvement due to their modular architecture, which allows plug-and-play integration of various components. Consequently, we classify carefully designed CNN models based on their primary features into fundamental models, including VGGNet, ResNet, GoogleNet, DenseNet, EfficientNet, and others. Improved models that use the same basic architecture often share similar properties and design principles.

#### 4.2.1. CNNs

VGGNet, proposed by the Visual Geometry Group at Oxford University in 2014, is widely adopted in brain age prediction studies spanning all age groups (N = 16). Initially applied to 2D image tasks [79], its adaptation to brain age prediction demonstrates its practicality. For instance, Hwang et al. utilized a 2D VGGNet model to validate the feasibility of predicting brain age across the entire age spectrum using slices of routine clinical T2 MRI slices [53]. Expanding VGGNet to 3D enhances its capability to process complex neuroimaging data, though researchers often use simpler, shallow architectures [19,48,50,52,55] to manage the increased data complexity. One notable adaptation is the simple fully convolutional network (SFCN), which integrates VGGNet principles with fully convolutional methods, achieving impressive results with a mean absolute error (MAE) of 2.14 years on the UKB dataset. Building on this, Leonardsen et al. introduced variants like SFCN-sm and SFCN-reg, demonstrating advancements in discrete and continuous age prediction [73]. Integrating multiple modalities further improves accuracy. Hofmann et al. proposed a multi-level ensembles approach [57], where the initial phase trains several VGGNets on various modalities and a linear head model on the validation set. The subsequent phase integrates the linear head models from different modalities to obtain the final prediction. Despite VGGNet’s effectiveness alone, ensemble models combining it with other architectures, like Siamese networks [46] and MLP models [63], highlight its role in advancing brain age prediction. Duchesne et al. used linear regression to integrate DL and ML algorithms at the decision level, including the best linear unbiased predictor, SVR, VGGNet, ResNet, and GoogleNet [58]. Similarly, Zhang et al. introduced the weighted ensemble, assigning weights to different models for different age groups, integrating VGGNet, ResNet, GoogleNet, and SVR [62]. These approaches provide valuable insights for high-performance brain age prediction across the entire age spectrum.

ResNet, introduced by He et al. in 2015 [80], addresses gradient issues and degradation with its residual structure, linking features across convolutional layers. In brain age prediction tasks spanning all ages, ResNet models are prevalent (N = 8). For instance, Hepp et al. utilized a 3D-extended ResNet with Grad-CAM to highlight brain subregions, like the ventricles, insular lobe, and basal ganglia, and the internal capsule relevance [61]. Ensembling multiple ResNet models enhances robustness. Kuo et al. fused predictions from five ResNet models using input-level fusion and decision-level fusion techniques, achieving high predictive performance [69]. Similarly, Ballester et al. employed ResNet18 with input-level fusion using dual-channel GM and WM slices and decision-level fusion across axial, coronal, and sagittal plane slices [49]. These ensemble strategies underscore the importance of carefully selecting derived phenotypes from single-modality data.

Introduced by Google in 2014, GoogleNet excelled in the 2014 ImageNet Large-Scale Visual Recognition Challenge with remarkable efficiency, using significantly fewer parameters than VGGNet16 [81]. Its standout feature, the inception module, employs diverse convolution kernel sizes to capture multi-scale perceptions. In brain age prediction tasks spanning all ages, GoogleNet or its design principles are widely adopted (N = 7). For instance, Bashyam et al. used GoogleNet (Inception-ResNet-v2) pre-trained on ImageNet for their DeepBrainNet approach [56], demonstrating its utility in linking brain age differences and cognitive function [20]. Kianian et al. utilized a variant of the inception architecture, Xception [60], which enhances computational efficiency for smaller images by restructuring the convolutional process [82]. This design makes Xception highly efficient for processing small-scale images. Their 2D CNN model, the greedy dual-stream, integrated both local and global image pathways. Extending GoogleNet to 3D poses computational and memory challenges. Wang et al. integrated atrous spatial pyramid pooling layers into a shallow architecture to balance computational demands with effective multi-scale information extraction [70]. Armanious et al. enhanced predictive performance by combining GoogleNet and SqueezeNet [77], leveraging SqueezeNet’s parameter efficiency alongside GoogleNet’s capabilities, achieving competitive results with an MAE of 1.955 years.

DenseNet, proposed by Huang et al. in 2017, builds on ResNet concepts with dense connectivity, ensuring direct links between all layers to enhance feature reuse and extraction [83] It has gained traction in brain age prediction studies across all ages. Wood et al. used a 3D DenseNet121 model [65], while Cheng et al. incorporated DenseNet’s dense connections into a two-stage network for brain age estimates [72].

EfficientNet, known for optimizing model size, depth, and resolution via compound scaling, achieves efficient performance and higher accuracy in image classification tasks [84]. Poloni et al. extended the EfficientNet to a 3D model for brain age prediction, using hippocampal region image blocks and employing a systematic two-stage transfer learning approach across different age groups [59]. The initial stage involved pre-training the model using image blocks from subjects aged 20–70 years. In the subsequent stage, they refined the pre-trained model by including image blocks from subjects over 70 years old.

Feedforward neural networks (FNNs) are characterized by their unidirectional flow of information, which progresses from the input layer, through any intermediate hidden layers, and culminates at the output layer. In the context of DL, the term is applied to FNNs that possess a substantial depth, consisting of multiple interconnected layers. Wu et al. employed an FNN to map data from a high-dimensional feature space to a low-dimensional embedding, subsequently using KNN regression to perform brain age prediction across the full age spectrum [41]. In contrast, Bellantuono et al. adopted a different methodology, extracting patches from preprocessed T1 MRI data. These patches were then vectorized and used to construct correlation matrices. The node strength, derived from these matrices, was utilized as a feature set for brain age prediction using an FNN [75].

The U-Net architecture, was specifically designed for the purpose of biomedical image segmentation [85]. This model has garnered significant attention due to its proficiency in capturing and maintaining finer-grained scale information, a feature that is particularly advantageous for tasks that require high-resolution analysis. In the realm of brain age prediction, Popescu et al. have showcased an innovative application of the U-Net model in their research [42].

Graph convolutional networks (GCNs), as a specialized category of CNNs, represent a pioneering paradigm in the field of deep learning [86]. The unique attribute of GCNs lies in their ability to seamlessly integrate with non-Euclidean data structures, which are prevalent in complex domains such as brain connectivity graphs [87]. Besson et al. have exemplified the application of GCNs by designing a model that is based on the ResNet architecture, achieving commendable model performance [47]. Additionally, Gopinath et al. proposed a novel graph pooling technique that is adept at effectively aggregating multiple surface-valued data [51].The core of this innovation is the method’s ability to synthesize graph nodes via a learned spectral graph embedding process. This process not only enriches the representational power of the model but also significantly enhances its predictive capabilities.

Two specialized networks are also reported in this review. Xu et al. utilized a Siamese network with node convolution (SNNC), a novel approach that deviates from the conventional single-sample input paradigm. By employing sample pairs as the input, the SNNC is adept at mitigating the challenge of insufficient sample sizes [44]. The results of their study demonstrated that SNNC is capable of delivering robust predictions even with a modest number of samples. Fu et al. proposed an optimal transport-based feature pyramid fusion (OTFPF) network, which has set a new benchmark in the accuracy of brain age prediction across the full age spectrum [78].

#### 4.2.2. CNNs with Transformers

In the quest for enhanced predictive models in the domain of brain age prediction, researchers have ingeniously merged the distinct capabilities of CNNs and Transformers. This strategic integration has culminated in a robust architecture framework that is adept at capturing both local and global information in complex datasets. A direct approach involves using a CNN-based model for feature extraction, which is then followed by a Transformer model for features fusion. He et al. exemplified this approach with a dual-pathway model, where VGGNet serves as the backbone architecture. Ingeniously designed global–local transformers are employed to integrate global contextual information and fine-grained local information [71]. Similarly, Chen et al. further advanced this concept with the development of the Segmentation-Transformer-Age-Network, a two-stage model aimed at improving brain age prediction accuracy. Initially, a U-Net-inspired segmentation model delineates brain structures. Subsequently, a segmentation Transformer amalgamates global and local features to enhance prediction accuracy [54]. Parallel to these developments, an alternative strategy involves the modular integration of the self-attention mechanism, inherent to Transformer models, into CNN architectures. This innovative approach represents a subtle yet powerful fusion of transformative elements. For instance, the FiA-Net model by He et al., with ResNet as its backbone, incorporates hierarchical fusion modules with attention mechanisms, significantly boosting model performance [64]. Likewise, Lim et al. introduced a multi-hop graph attention (MGA) module that constructs local and global connections using image features extracted by CNNs [68]. Additionally, Zhang et al. effectively integrated anatomical features extracted from DL modal models with imaging-based feature sets through an anatomy feature attention (AFA) module [74].

## 5. Discussion

With the growing interest of researchers in the field of brain age prediction across the entire age spectrum, a wide range of ML and DL models have been adopted and optimized, yielding commendable results. However, it must be acknowledged that brain age prediction based on neuroimaging data across the full age spectrum still faces ongoing and complex challenges. In the following discussion, we analyze and elucidate several key aspects, including model architectures and dataset construction, among other significant dimensions. These aspects collectively highlight the complexity of the brain age prediction task across the entire age spectrum, underscoring the necessity for innovation and continuous research to overcome the persistent challenges in this field.

### 5.1. ML vs. DL

Neuroimaging data are renowned for their inherent richness of information. Traditional ML methods typically require manual feature extraction, a process fraught with complexity and variability in predictive efficacy based on the engineered features [36]. This complexity has driven the exploration of various ML approaches to better harness the potential of neuroimaging data. For ML methods, although parametric ML models are simple to construct and can significantly simplify the learning process, they assume prior knowledge about the data distribution [36,38,39]. This assumption makes them less capable of handling complex data models and prone to large biases when the training data are insufficient. Therefore, researchers tend to prefer non-parametric ML models for the complex task of brain age prediction across the entire age spectrum. However, this flexibility comes at the cost of higher computational complexity and the necessity for large datasets to fully exploit their advantages. For example, Kalc et al. used a GPR model and achieved very high predictive performance on the UKB database [40]. The substantial size of the UKB dataset allowed the GPR model to effectively learn and generalize from the data, demonstrating its potential in handling complex neuroimaging information. In contrast, Ly et al., who also used a GPR model for brain age prediction, encountered relatively poor predictive performance due to the smaller dataset they employed [37]. This disparity underscores the importance of dataset size in the efficacy of non-parametric models. Furthermore, Han et al. [38] and Lee et al. [39] conducted performance comparison experiments of various ML methods, providing additional insights into the dynamics between parametric and non-parametric models. Their studies revealed that when the dataset is relatively small, parametric and non-parametric ML models exhibit similar predictive performances. This finding suggests that the advantages of non-parametric models become more pronounced with larger datasets, highlighting the critical role of data volume in achieving superior predictive accuracy.

The emergence of DL, particularly CNNs, has revolutionized this landscape by automating feature extraction. This technological leap empowers researchers to process and analyze neuroimaging data more efficiently. However, despite the tremendous potential demonstrated by DL methods, the decision on whether to choose ML or DL approaches in practical applications remains inconclusive.

**Figure 4:**
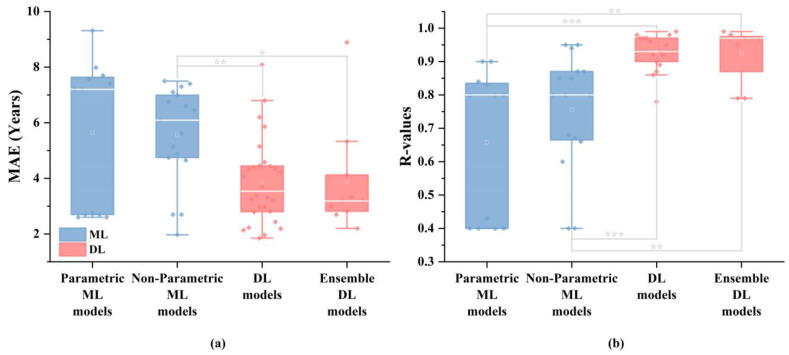
Model performance differences between DL and ML methods. The white line represents the median, and the white square indicates the mean. Each data point in the figure represents the performance of a model on the test set. SPSS Mann–Whitney U test was used to analyze the differences between each group; * represents p<0.05, ** represents p<0.01; *** represents p<0.001. (**a**) exhibits MAE for different types of models, (**b**) exhibits *R-values* for different types of models.

Figure 4 illustrates marked performance disparities between ML and DL methods in this review. The performance comparison included the performance results of 29 different models reported in 11 studies that utilized ML models (with multiple models used in the studies by Tesli et al. [33], Han et al. [1], More et al. [36] and Lee et al. [39]), as well as the performance results of 38 different models from 38 studies that employed DL models. Each data point in the figure represents the performance of a model on the test set. To assess the significance of the differences between groups, the Mann–Whitney U test was performed for pairwise comparisons among the four subgroups: parametric ML models, non-parametric ML models, DL models, and ensemble DL models. Additionally, the Mann–Whitney U test was also conducted between the ML model performances (parametric ML models and non-parametric ML models) and the DL model performances (DL models and ensemble DL models). In terms of MAE, DL methods exhibit significantly superior results compared to those of the ML methods (p=0.003). Similarly, for *R-values*, the DL methods also outperform the ML methods (p<0.001). These findings underscore DL’s efficacy in accurately predicting brain age across the entire age spectrum. However, it must be acknowledged that DL models, especially CNNs, typically require more computational power compared to ML models due to their complex architecture and large number of parameters [88].

**Figure 5:**
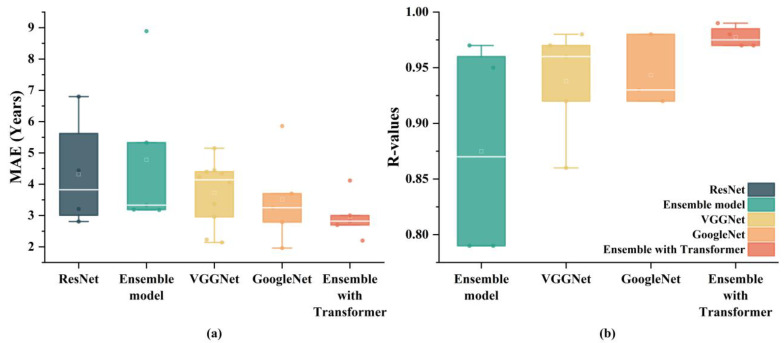
The performance differences among various DL models. The white line represents the median, and the white square indicates the mean. Each data point in the figure represents the performance of a model on the test set. (**a**) exhibits MAE for different types of DL models, (**b**) exhibits *R-values* for different types of DL models. Due to the limited reporting of *R-values* for ResNet, the boxplot does not include ResNet.

For DL models, different model architectures exhibit varying levels of performance. Figure 5 illustrates a comparison of several commonly used DL models for brain age prediction tasks across the entire age spectrum (N = 38). Each data point in the figure represents the performance of a model on the test set. The architecture of a DL model significantly impacts its performance, as evidenced by the results. Simple architectures like VGGNet demonstrate superior performance compared to more advanced architectures like ResNet. One reason VGGNet performs better is due to its relatively straightforward architecture. This structure allows VGGNet to capture hierarchical features effectively without being overly complex. Additionally, the simplicity of VGGNet facilitates more efficient training and better generalization to unseen data. GoogleNet outperforms other individual models, including VGGNet. GoogleNet’s success can be attributed to its inception modules. These modules allow the model to perform convolutions with multiple filter sizes simultaneously, capturing a variety of feature scales and leading to a richer representation of the data. While ensemble models generally aim to leverage the strengths of multiple models, the basic ensemble model does not outperform individual models like VGGNet and GoogleNet. This finding suggests that the manner in which the models are combined plays a critical role in the success of ensemble approaches. A basic ensemble might not sufficiently integrate the complementary strengths of its constituent models, leading to suboptimal performance. In contrast, the superior performance of models integrated with Transformers highlights their capability to handle complex patterns in data more effectively. Their attention mechanisms enable them to focus on relevant parts of the input data, capturing long-range dependencies and intricate patterns that other models might miss. This makes Transformer-integrated models particularly suitable for tasks involving high-dimensional data like neuroimaging.

Post hoc interpretability of inference processes in brain age prediction models across the full age spectrum aids researchers in understanding the key factors influencing model decisions and the trajectories of brain development and aging. Compared to DL models, ML models generally offer greater transparency. For highly interpretable models like RF or DT, the importance of features can be assessed by estimating each feature’s impact on prediction performance, as demonstrated by Engemann et al. [32]. For more complex ML models, post hoc interpretability methods such as SHapley Additive exPlanations (SHAP) provide valuable insights. For instance, Ballester et al. explored the interactions between SHAP scores for each feature and group as a function of brain age gap, identifying total gray matter volume as the most significant feature in predicting brain age for patients with schizophrenia [34]. Similarly, Han et al. found that in predicting brain age in healthy subjects, the most important features included total intracranial volume, cortical thickness in frontal regions (superior frontal gyrus, caudal middle frontal gyrus, and pars triangularis), parietal regions (precuneus and supramarginal gyrus), and the surface area of regions in the superior frontal gyrus, lateral orbitofrontal gyrus, and middle temporal gyrus [38]. Due to the larger number of parameters in DL models, post hoc interpretability is more challenging compared to ML models. Class activation mapping (CAM) and its derivatives are among the most commonly used techniques for interpreting DL models in brain age prediction tasks across the full age spectrum, as demonstrated in the studies by Besson et al. [47], Feng et al. [55], Zhang et al. [74], Gautherot et al. [52], and Hepp et al. [61]. Additionally, Hofmann et al. applied the layer-wise relevance propagation (LRP) algorithm and identified pronounced contributions from voxels surrounding the ventricles and at the borders [57]. Wood et al. [65] and Lee et al. [39] employed occlusion sensitivity analysis to interpret their models. It is worth noting that some novel visualization techniques differ from traditional methods. For instance, He et al. designed a global–local attention mechanism model that uses patches with optimal MAE performance to construct heatmaps, capturing subtle neuroanatomical changes associated with different age groups [71]. He et al. discovered that in children aged 0–5 years, the model focused more on the frontal lobe, transitioned to the deep gray matter regions in children aged 5–20 years, gradually shifted its focus to the parietal lobe between ages 30–35, then returned to the frontal lobe at ages 35–40, and then shifted back at 35–40 years until 65–70 years [71].

### 5.2. Construction of a Neuroimaging Dataset Spanning the Entire Age Spectrum

Creating a comprehensive dataset that spans the entire age spectrum is a challenging endeavor due to the inherent difficulty in obtaining a large volume of neuroimaging data across all age groups. Typically, such datasets are constructed by amalgamating several neuroimaging datasets, each focusing on different age segments. This approach helps to cover the full range from early childhood to old age, enabling researchers to study the brain’s development and aging processes comprehensively. Pediatric datasets provide crucial information on brain development during early years, capturing rapid growth and significant changes in brain structure and function. These datasets are vital for understanding the foundational stages of neural development. Adolescent datasets, on the other hand, offer insights into the significant changes occurring during puberty, a critical period characterized by brain maturation and reorganization. Understanding these changes is essential for studying the impact of puberty on cognitive and emotional development. Adult datasets are essential for understanding the stable phase of brain function, where major developmental changes have subsided, and the brain’s structure and function are relatively stable. These datasets help researchers explore the neural basis of cognitive abilities, mental health, and the effects of various environmental factors on the brain. Elderly datasets are critical for studying age-related cognitive decline and neurodegenerative diseases. They provide invaluable data for investigating the progression of conditions such as Alzheimer’s disease (AD) and other dementias, as well as understanding the normal aging process. By integrating these diverse datasets, researchers can create a more holistic view of brain aging across the entire human lifespan. However, this method also introduces challenges related to data harmonization, variability in imaging protocols, and demographic differences, which must be carefully managed to ensure the reliability and validity of the combined dataset.

Extensive publicly available neuroimaging databases facilitate brain age prediction tasks across the entire age spectrum. One of the most commonly used databases is the IXI dataset (N = 24), which contains multimodal neuroimaging data, including T1 MRI, T2 MRI, MRA, and dMRI, from approximately 600 healthy subjects aged 19 to 87. This database is particularly notable for its ease of access and broad age range, making it a valuable resource for researchers. Another significant resource is the Cam-CAN (N = 16) database, a large-scale collaborative research project by the University of Cambridge, which aims to use epidemiological, cognitive, and neuroimaging data to understand how individuals can best retain cognitive abilities into old age. It includes MRI, fMRI, MEG, and various cognitive experiment data from nearly 700 healthy subjects aged 18 to 88, providing a comprehensive view of brain function across different age groups. The ADNI database is also widely used in brain age prediction tasks across the full age spectrum (N = 13). This database aims to explore the progression of AD and includes MRI and PET images, genetic data, and cognitive test results from AD subjects, individuals with mild cognitive impairment (MCI), and healthy controls, aged 50 to 97.

**Figure 6:**
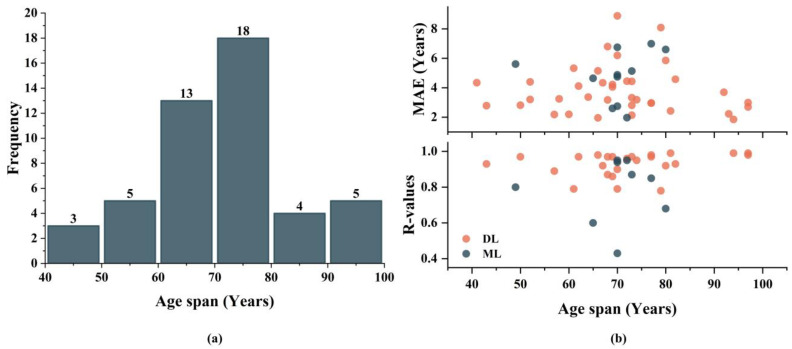
Summary of different study age spans and model performances. (**a**) exhibits the frequency of studies across different age spans, (**b**) exhibits model performances in studies across different age spans.

Thanks to these publicly available neuroimaging datasets, researchers have constructed personalized study cohorts by integrating multiple datasets, resulting in cohorts spanning ages 41 to 97. Figure 6a shows the distribution of studies across various age ranges, with the majority of studies focusing on an age span between 60 and 80. Figure 6b displays the model prediction performance across different age spans. In regression tasks, model performance generally decreases with broader age spans. We calculated the Spearman correlation coefficient between age span and model performance, revealing no significant correlation between subject age span and prediction accuracy. This suggests that while models across all age groups exhibit some variability in age spans, their performances remain comparable.

**Figure 7:**
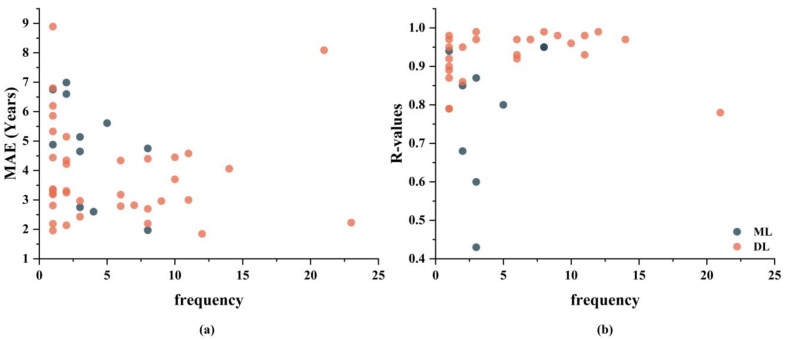
Performance of models using different numbers of databases. (**a**) exhibits MAE of models using different numbers of databases, (**b**) exhibits *R-values* of models using different numbers of databases.

Due to the varying collection protocols and equipment used across different publicly available neuroimaging datasets, the study cohorts constructed from multiple datasets exhibit heterogeneity. This heterogeneity can potentially enhance model generalization [73,78], but it might also make it more challenging for models to learn patterns [48]. The inconsistency in neuroimages can make it more difficult for ML or DL algorithms to identify and learn the underlying relationships and patterns. Figure 7 shows the performances of models when using different numbers of datasets. The results indicate that for ML models, the MAE significantly decreases as the number of datasets used increases (Spearman correlation: *p* = 0.0423, *R-values* = −0.6190), although there is no significant improvement in *R-values*. Conversely, this trend is not observed in DL models. This suggests that utilizing a greater diversity of heterogeneous data presents greater challenges for brain age prediction models, particularly those based on ML. However, DL models, owing to their superior feature learning capabilities, are better equipped to address these challenges to some extent. To enhance model generalization performance, it is advisable to integrate a broader array of datasets when constructing the dataset.

Leonardsen et al. assembled a study cohort from 23 publicly available neuroimaging datasets, including T1 MRI data from 56,095 subjects aged 3 to 96. This represents the largest to date for brain age prediction tasks across the entire age spectrum, encompassing nearly all commonly used public neuroimaging datasets, such as IXI, ADNI, Cam-CAN, UKB, and ABIDE. The models trained on this cohort demonstrated highly competitive predictive performance [73]. In contrast, Popescu et al. complied a study cohort from 21 public neuroimaging datasets, establishing it as the second most heterogeneous dataset for brain age prediction tasks across the entire age spectrum. However, this cohort included only T1 MRI data from 3873 subjects aged 18 to 97. Due to the high heterogeneity and smaller dataset size, the models exhibited suboptimal performance [42]. This underscores the importance of careful consideration when constructing heterogeneous datasets. Additionally, Feng et al. collected over 30,000 T1 MRI datasets from multiple open neuroimaging sources, spanning ages 18 to 97. They applied a balanced sampling approach incorporating both “oversampling” and “under-sampling” techniques, providing insights into the addressal of potential dataset imbalance issues [55]. Few public datasets cover the entire age spectrum, while there are more datasets focusing on adolescent brain development and elderly brain aging. This often results in study cohorts with a higher proportion of adolescent and elderly subjects and a relative scarcity of middle-aged subjects, leading to imbalances [64,71,73]. To address potential issues arising from age imbalance, the sampling approach used by Feng et al. is a commonly employed method [55]. However, it is worth noting that repeated data might introduce new potential problems. It is also noteworthy that different racial groups often exhibit variations in brain structure. Existing public neuroimaging datasets make it challenging to construct racially balanced datasets. For instance, the IXI dataset primarily consists of white individuals, and the ADNI dataset has limited representation of non-white individuals [89]. Similar racial biases are also present in the UKB dataset [90]. Models trained on such datasets for the entire age spectrum raise concerns about generalizability. Addressing this challenge requires close international collaboration to develop models that generalize robustly across different cultural and genetic backgrounds [91]. It is noteworthy that external validation across diverse populations and geographic regions can help ensure the broad applicability of models. Furthermore, the trajectories of brain development and aging differ between sexes. Therefore, differences in sex distribution within training data may reduce the generalizability of models. To mitigate potential sex distribution bias in brain age prediction across the entire age spectrum, researchers often thoughtfully include sex as a feature in model inputs [63].

### 5.3. Challenges and Future Directions

Multimodal Data Integration: Due to the requirement for large datasets that span the full age spectrum, most existing studies have predominantly relied on single-modality T1-weighted MRI data. To achieve a more comprehensive and accurate estimation of brain age, it is crucial to integrate multiple imaging modalities. This approach would likely offer deeper insights into the biomarkers associated with brain development and aging trajectories, ultimately leading to a more holistic understanding of brain aging across different life stages.

Exploration of DL Model Architectures: As highlighted in our literature review, DL models have become a focal point in this field due to their superior performance compared to ML models. Although the introduction of Transformer models has invigorated the development and exploration of DL models, research on DL architectures in this domain is still in its early stages. Improved Transformer models, such as Retentive Network, Reformer, and Linformer, have yet to be thoroughly explored. Additionally, there is a lack of comprehensive studies that benchmark the performance of various DL architectures using unified datasets and evaluation metrics.

Model Interpretability: ML models, especially DL models, inherently possess “black box” characteristics, meaning the reasoning and decision-making processes of these models are often opaque. Moreover, in the complex task of brain age prediction across the full age spectrum, models with transparent algorithms tend to underperform compared to DL models. Research on the interpretability of DL models is limited. Therefore, future studies should incorporate post hoc interpretability techniques. This approach could not only help uncover novel biomarkers related to brain development and aging but could also inform decisions that may impact patient outcomes.

## 6. Conclusions

Research indicates that DL methods significantly outperform ML methods. Among DL methods, the strategic integration of Transformer models within DL frameworks demonstrates the highest overall model performance. Constructing comprehensive age-spectrum study cohorts is a critical step in brain age prediction tasks. The results suggest that the age span does not significantly impact model performance, while including a larger number of subjects subtly enhances model performance. While increasing data heterogeneity increases the difficulty of model learning, it also improves model generalization. When constructing age-spectrum study cohorts, it is essential to consider the balance of age, race, and gender. Future efforts should prioritize establishing balanced and heterogeneous study cohorts, designing more advanced DL model architectures and collectively focusing on enhancing predictive insights into the complex processes of brain development and aging.

## Figures and Tables

**Table 1:** Summaries of brain age prediction tasks across the entire age spectrum using ML algorithms.

Reference	Dataset	Age Range	Age Span	Subject	Data Modality	Models	*MAE* (years)	*R-Values*
Beck et al. [31]	TOP, StrokeMRI	18–95	77	702	dMRI	DT model to stack XGBoost	6.99	0.85
Engemann et al. [32]	CamCAN	18–88	70	674	T1 MRI, fMRI, Magnetoencephalography (MEG)	RF model to stack RR	6.75	-
Tesli et al.* [33]	TOP, StrokeMRI	12–92	80	586	T1 MRI	RF, XGBoost	6.6	0.68
Ballester et al. [34]	COBRE, MCIC, UCLA, TOPSY, CAN-BIND	16–65	49	471	fMRI	XGBoost	5.61	0.8
Han et al.* [1]	Simulated dataset, CoRR, NKI	12–85	73	125	fMRI	SVR, RVR, Lasso, EN, RR, XGBoost	5.14	0.87
Xifra-Porxas et al. [35]	CamCAN	18–88	70	613	T1 MRI, MEG	RF model to stack GRP	4.88	0.94
More et al.* [36]	CamCAN, IXI, eNKI, 1000 Brains, CoRR, OASIS-3, MyConnectcome, ADNI	18–88	70	2953	T1 MRI	RR, Lasso, EN, and 5 other ML methods	4.75	0.95
Ly et al. [37]	ADNI, IXI, OASIS-3	20–85	65	1248	T1 MRI	GPR	4.65	0.6
Han et al.* [38]	HCP, CamCAN, IXI	18–88	70	2281	T1 MRI	Lasso, RR, EN, and 24 other ML methods	2.75	0.43
Lee et al.* [39]	HCP, CamCAN, ISMMS, COBRE	18–87	69	1584	T1 MRI	RR, Lasso, EN, and 3 other ML methods	2.6	-
Kalc et al. [40]	UKB, IXI, OASIS-3, Cam-CAN, SALD, NKI, ADNI, SZ samples	18–90	72	40,070	T1 MRI	GRP	1.97	0.95

* indicates that only the performance of the optimal model in the article is shown in table. The absence of *R-values* indicates that the performance metric *R-values* were not reported in this study. The full names of the datasets are as follows: TOP: the Tematisk Område Psykoser; CamCAN: Cambridge Centre for Ageing and Neuroscience; COBRE: the Center for Biomedical Research Excellence; MCIC: the schizophrenic and matched control; UCLA: the UCLA Consortium for Phenomics; TOPSY: the Tracking Outcome in Psychosis; CAN-BIND: the Canadian Biomarker Network for Depression; CoRR: Consortium for Reliability and Reproducibility; NKI: Nathan Kline Institute; IXI: Information eXtraction from Images; OASIS: The Open Access Series of Imaging Studies; ADNI: Alzheimer’s Disease Neuroimaging Initiative; HCP: Human Connectome Project; ISMMS: the Icahn School of Medicine at Mount Sinai; UKB: UK Biobank; SALD: Southwest University Adult Lifespan; SZ: schizophrenia patients.

**Table 2:** Summaries of brain age prediction tasks across the entire age spectrum using DL algorithms.

Reference	Dataset	Age Range	Age Span	Subject	Data Modality	Models	*MAE* (years)	*R-Values*
Wu et al. [41]	CamCAN	18–88	70	600	fMRI	Ensemble model (FNN, KNN)	8.89	0.79
Popescu et al. [42]	ABIDE, Beijing Normal University, Berlin School of Brain Mind, CADDementia, Cleveland Clinic, ICBM, IXI, MCIC, MIRIAD, NEO2012, NKI, OASIS, WUS, TRAIN-39, BAHC, DLBS, CamCAN, SALD, Wayne state, OASIS-3, AIBL	18–97	79	3873	T1 MRI	3D U-Net	8.09	0.78
Borkar et al. [43]	CamCAN	20–88	68	638	fMRI	2D ResNet	6.8	0.87
Xu et al. [44]	CamCAN	18–88	70	600	fMRI	2D Siamese Network	6.2	0.9
Valdes-Hernandez et al. [45]	UF Health System	15–95	80	1559	T1 MRI, T2 MRI	2D GoogleNet	5.86	0.92
Ding et al. [46]	SLIM	19–80	61	494	fMRI	3D Ensemble model (SFCN, Siamese network)	5.33	0.79
Pardakhti et al. [19]	IXI, ADNI-I	20–86	66	609	T1 MRI	3D VGGNet	5.15	-
Besson et al. [47]	ABIDE II, Age-ility, CamCan, CoRR, DLBS, BGSP, HCP, IXI, MPI-LMBB, NKI, SALD	7–89	82	6410	T1 MRI	GCN	4.58	0.93
Cheng et al. [48]	IXI, SALD, NKI, CoRR, UKB, PNC, 973, HCP, Organ Transplantation Center, Tianjin First Central Hospital	8–80	72	3743	T1 MRI	3D VGGNet	4.45	0.96
Ballester et al. [49]	PAC2019	17–90	73	3298	T1 MRI	2D ResNet	4.44	-
Kuchcinski et al. [50]	IXI, HCP, OBRE, MCIC, NMorphCH, NKI-RS, PPMI, ADNI	18–70	52	1503	T1 MRI	3D VGGNet	4.4	-
Gopinath et al. [51]	Mindboggle-101, ADNI-I	20–61	41	101	T1 MRI	GCN	4.35	-
Gautherot et al. [52]	IXI, HCP, COBRE, MCIC, NMorphCH, NKI-RS	18–85	67	2065	T1 MRI	3D VGGNet	4.34	0.92
Hwang et al. [53]	Seoul National University Hospital, IXI	19–88	69	2360	T2 MRI	2D VGGNet	4.22	0.86
Chen et al. [54]	-	18–80	62	712	T1 MRI, Quantitative susceptibility mapping (QSM)	3D U-Net with Transformer	4.12	0.97
Feng et al. [55]	ADNI, AIBL, NIFD, IXI, BGSP, OASIS-1, OASIS-2, SALD, SLIM, PPMI, SchizConnect, DLBS, CoRR, CamCAN	18–97	69	6794	T1 MRI	3D VGGNet	4.06	0.97
Bashyam et al. [56]	ADC, AIBL, BLSA, CARDIA, GAP, PAC, PING, PNC, PennPMC, SHIP	3–95	92	14,468	T1 MRI	2D GoogleNet	3.7	-
Hofmann et al. [57]	The LIFE Adult study	18–82	64	2016	T1 MRI, susceptibility-weighted magnitude images (SWI), Fluid-attenuated inversion recovery images (FLAIR)	3D VGGNet	3.37	-
Duchesne et al. [58]	PAC2019	17–90	73	2640	T1 MRI	3D Ensemble model (Best Linear Unbiased Predictor, SVR, VGGNet, ResNet, and GoogleNet)	3.33	-
Poloni et al. [59]	IXI, ADNI	min = 20, max > 70	-	1189	T1 MRI	3D EfficientNet	3.31	0.95
Kianian et al. [60]	IBID, IXI	19–77	58	869	T1 MRI	2D XceptionNet	3.25	-
Hepp et al. [61]	GNC	20–72	52	10,691	T1 MRI	3D ResNet	3.21	-
Zhang et al. [62]	PAC2019	16–90	74	2641	T1 MRI	3D Ensemble model (VGGNet, ResNet, GoogleNet, SVR)	3.19	0.95
Joo et al. [63]	FCP1000, INDI, IXI OASIS-3, OpenNeuro, CamCAN	18–86	68	3004	T1 MRI	3D Ensemble model (VGGNet, Multi-layer Perception)	3.18	0.97
He et al. [64]	MGHBCH, NIH-PD, ABIDE-I, BGSP, BeijingEN, IXI, DLBS, OASIS-3, ABCD, HBN, CoRR	0–97	97	16,705	T1 MRI	3D ResNet with Transformer	3	0.98
Wood et al. [65]	KCH, GSTT, IXI	18–95	77	23,865	T1 MRI, T2 MRI, diffusion-weighted images (DWI)	3D DenseNet	2.97	0.97
Dular et al. [66,67]	ABIDE, ADNI, CamCAN. CC-359, FCP1000, IXI, OASIS-2, UKB, OASIS-1	18–95	77	4313	T1 MRI	3D VGGNet	2.96	0.98
Lim et al. [68]	OpenNeuro, COBRE, OpenfMRI, INDI, IXI, FCP1000, XNAT	20–70	50	2788	T1 MRI	3D ResNet with Transformer	2.82	0.97
Kuo et al. [69]	PAC2019	17–90	73	3143	T1 MRI, T2 MRI	3D ResNet	2.81	0.97
Wang et al. [70]	COBRE, Beijing-Enhanced, CamCAN, HCP, SLIM, PPMI	17–60	43	2406	DTI	3D GoogleNet	2.79	0.93
He et al. [71]	BGSP, OASIS-3, NIH-PD, ABIDE-I, IXI, DLBS, HBN, CoRR	0–97	97	8379	T1 MRI	2D VGGNet with Transformer	2.7	0.99
Cheng et al. [72]	OASIS, ADNI-1, PAC2019	17–98	81	6586	T1 MRI	3D DenseNet	2.43	0.99
Leonardsen et al. [73]	HBN, ADHD200, PING, ABIDE, SLIM, ABIDE-2, Beijing, AOMIC, CoRR, MPI-LMBB, HCP, FCP1000, NKI, IXI, Oslo, ADNI, AIBL Roc-land, SALD, DLBS, CamCAN, UKB, OASIS-3, OpenNeuro	3–96	93	56,095	T1 MRI	3D SFCN	2.23	-
Zhang et al. [74]	FCP1000, ADNI, DLBS, IXI, NRTC, OASIS, PPMI, SALD	20–80	60	2382	T1 MRI	3D VGGNet with Transformer	2.2	-
Bellantuono et al. [75]	ABIDE	7–64	57	1016	T1 MRI	FNN	2.19	0.89
Peng et al. [76]	UKB, PAC2019	17–90	73	17,801	T1 MRI	3D SFCN	2.14	-
Armanious et al. [77]	IXI	20–86	66	562	T1 MRI	3D GoogleNet	1.96	0.98
Fu et al. [78]	ABIDE I, ABIDE II, ADNI, BGSP, CoRR, DLBS, ICBM, IXI, NKI, OASIS-3, OpenfMRI, SALD	3–97	94	12,909	T1 MRI	3D OTFPF	1.85	0.99

The absence of *R-values* indicates that the performance metric *R-values* were not reported in this study. The full names of the datasets are as follows: ABIDE: the Autism Brain Imaging Data Exchange; CADDementia: Computer-Aided Diagnosis of Dementia; ICBM: International Consortium for Brain Mapping; MIRIAD: Minimal Interval Resonance Imaging in Alzheimer’s Disease; BAHC: Brain-Age Healthy Controls; DLBS: Dallas Lifespan Brain Study; AIBL: Australian Imaging Biomarkers and Lifestyle Study of Aging; SLIM: Southwest University Longitudinal Imaging Multimodal; BGSP: Brain Genomics Superstruct Project; MPI-LMBB: MPI-Leipzig Mind-Brain-Body; PNC: Philadelphia Neurodevelopmental Cohort; OBRE: Center of Biomedical Research Excellence; NMorphCH: Neuromorphometry by Computer Algorithm Chicago; PPMI: Parkinson’s Progression Markers Initiative; BLSA: Baltimore Longitudinal Study of Aging; CARDIA: Coronary Artery Risk Development in Young Adults; GNC: the German National Cohort Study; MGHBCH: Massachusetts General and Boston Children’s Hospitals; NIH-PD: NIH-Pediatric Data; ABCD: The Adolescent Brain Cognitive Development; HBN: Healthy Brain Network; KCH: King’s College Hospital NHS Foundation Trust; GSTT: Guy’s and St Thomas’ NHS Foundation Trust; CC-359: Calgary-Campinas-359; FCP1000: 1000 Functional Connectomes Project; INDI: International Neuroimaging Data-sharing Initiative; ADHD200: Attention Deficit Hyperactivity Disorder; PING: Pediatric Imaging, Neurocognition, and Genetics; AOMIC: The Amsterdam Open MRI Collection.

## Data Availability

Not applicable.

## References

[1] Han H., Ge S., Wang H. (2023). Prediction of Brain Age Based on the Community Structure of Functional Networks. Biomed. Signal Process. Control.

[2] Tanveer M., Ganaie M.A., Beheshti I., Goel T., Ahmad N., Lai K.-T., Huang K., Zhang Y.-D., Del Ser J., Lin C.-T. (2023). Deep Learning for Brain Age Estimation: A Systematic Review. Inf. Fusion.

[3] Chang Y., Thornton V., Chaloemtoem A., Anokhin A.P., Bijsterbosch J., Bogdan R., Hancock D.B., Johnson E.O., Bierut L.J. (2024). Investigating the Relationship Between Smoking Behavior and Global Brain Volume. Biol. Psychiatry Glob. Open Sci..

[4] Daviet R., Aydogan G., Jagannathan K., Spilka N., Koellinger P.D., Kranzler H.R., Nave G., Wetherill R.R. (2022). Associations between Alcohol Consumption and Gray and White Matter Volumes in the UK Biobank. Nat. Commun..

[5] Hautasaari P., Savić A.M., Loberg O., Niskanen E., Kaprio J., Kujala U.M., Tarkka I.M. (2017). Somatosensory Brain Function and Gray Matter Regional Volumes Differ According to Exercise History: Evidence from Monozygotic Twins. Brain Topogr..

[6] de Manzano Ö., Ullén F. (2018). Same Genes, Different Brains: Neuroanatomical Differences Between Monozygotic Twins Discordant for Musical Training. Cereb. Cortex.

[7] Levine M.E. (2013). Modeling the Rate of Senescence: Can Estimated Biological Age Predict Mortality More Accurately Than Chronological Age?. J. Gerontol. Ser. A.

[8] Singh N.M., Harrod J.B., Subramanian S., Robinson M., Chang K., Cetin-Karayumak S., Dalca A.V., Eickhoff S., Fox M., Franke L. (2022). How Machine Learning Is Powering Neuroimaging to Improve Brain Health. Neuroinformatics.

[9] Cole J.H. (2020). Multimodality Neuroimaging Brain-Age in UK Biobank: Relationship to Biomedical, Lifestyle, and Cognitive Factors. Neurobiol. Aging.

[10] Franke K., Ziegler G., Klöppel S., Gaser C. (2010). Estimating the Age of Healthy Subjects from T1-Weighted MRI Scans Using Kernel Methods: Exploring the Influence of Various Parameters. NeuroImage.

[11] Xiong M., Lin L., Jin Y., Kang W., Wu S., Sun S. (2023). Comparison of Machine Learning Models for Brain Age Prediction Using Six Imaging Modalities on Middle-Aged and Older Adults. Sensors.

[12] Elliott M.L., Belsky D.W., Knodt A.R., Ireland D., Melzer T.R., Poulton R., Ramrakha S., Caspi A., Moffitt T.E., Hariri A.R. (2021). Brain-Age in Midlife Is Associated with Accelerated Biological Aging and Cognitive Decline in a Longitudinal Birth Cohort. Mol. Psychiatry.

[13] Jawinski P., Markett S., Drewelies J., Düzel S., Demuth I., Steinhagen-Thiessen E., Wagner G.G., Gerstorf D., Lindenberger U., Gaser C. (2022). Linking Brain Age Gap to Mental and Physical Health in the Berlin Aging Study II. Front. Aging Neurosci..

[14] Berger I., Slobodin O., Aboud M., Melamed J., Cassuto H. (2013). Maturational Delay in ADHD: Evidence from CPT. Front. Hum. Neurosci..

[15] Silva C.C.V., El Marroun H., Sammallahti S., Vernooij M.W., Muetzel R.L., Santos S., Jaddoe V.W.V. (2021). Patterns of Fetal and Infant Growth and Brain Morphology at Age 10 Years. JAMA Netw. Open.

[16] Lin L., Zhang G., Wang J., Tian M., Wu S. (2021). Utilizing Transfer Learning of Pre-Trained AlexNet and Relevance Vector Machine for Regression for Predicting Healthy Older Adult’s Brain Age from Structural MRI. Multimed. Tools Appl..

[17] Lin L., Jin C., Fu Z., Zhang B., Bin G., Wu S. (2016). Predicting Healthy Older Adult’s Brain Age Based on Structural Connectivity Networks Using Artificial Neural Networks. Comput. Methods Programs Biomed..

[18] Varzandian A., Razo M.A.S., Sanders M.R., Atmakuru A., Di Fatta G. (2021). Classification-Biased Apparent Brain Age for the Prediction of Alzheimer’s Disease. Front. Neurosci..

[19] Pardakhti N., Sajedi H. (2020). Brain Age Estimation Based on 3D MRI Images Using 3D Convolutional Neural Network. Multimed. Tools Appl..

[20] Jirsaraie R.J., Kaufmann T., Bashyam V., Erus G., Luby J.L., Westlye L.T., Davatzikos C., Barch D.M., Sotiras A. (2023). Benchmarking the Generalizability of Brain Age Models: Challenges Posed by Scanner Variance and Prediction Bias. Hum. Brain Mapp..

[21] Soumya Kumari L.K., Sundarrajan R. (2024). A Review on Brain Age Prediction Models. Brain Res..

[22] Seitz-Holland J., Haas S.S., Penzel N., Reichenberg A., Pasternak O. (2024). BrainAGE, Brain Health, and Mental Disorders: A Systematic Review. Neurosci. Biobehav. Rev..

[23] Muksimova S., Umirzakova S., Mardieva S., Cho Y.-I. (2023). Enhancing Medical Image Denoising with Innovative Teacher–Student Model-Based Approaches for Precision Diagnostics. Sensors.

[24] Khadse V.M., Mahalle P.N., Shinde G.R. (2020). Statistical Study of Machine Learning Algorithms Using Parametric and Non-Parametric Tests: A Comparative Analysis and Recommendations. Int. J. Ambient Comput. Intell. IJACI.

[25] Cole J.H., Ritchie S.J., Bastin M.E., Valdés Hernández M.C., Muñoz Maniega S., Royle N., Corley J., Pattie A., Harris S.E., Zhang Q. (2018). Brain Age Predicts Mortality. Mol. Psychiatry.

[26] Valizadeh S.A., Hänggi J., Mérillat S., Jäncke L. (2017). Age Prediction on the Basis of Brain Anatomical Measures. Hum. Brain Mapp..

[27] Friedman J.H. (2001). Greedy Function Approximation: A Gradient Boosting Machine. Ann. Stat..

[28] Chen T., Guestrin C. (2016). XGBoost: A Scalable Tree Boosting System. Proceedings of the 22nd ACM SIGKDD International Conference on Knowledge Discovery and Data Mining.

[29] Xu X., Lin L., Sun S., Wu S. (2023). A Review of the Application of Three-Dimensional Convolutional Neural Networks for the Diagnosis of Alzheimer’s Disease Using Neuroimaging. Rev. Neurosci..

[30] Vaswani A., Shazeer N., Parmar N., Uszkoreit J., Jones L., Gomez A.N., Kaiser Ł., Polosukhin I. (2017). Attention Is All You Need. Proceedings of the Advances in Neural Information Processing Systems.

[31] Beck D., de Lange A.-M.G., Maximov I.I., Richard G., Andreassen O.A., Nordvik J.E., Westlye L.T. (2021). White Matter Microstructure across the Adult Lifespan: A Mixed Longitudinal and Cross-Sectional Study Using Advanced Diffusion Models and Brain-Age Prediction. NeuroImage.

[32] Engemann D.A., Kozynets O., Sabbagh D., Lemaître G., Varoquaux G., Liem F., Gramfort A. (2020). Combining Magnetoencephalography with Magnetic Resonance Imaging Enhances Learning of Surrogate-Biomarkers. eLife.

[33] Tesli N., Bell C., Hjell G., Fischer-Vieler T., I Maximov I., Richard G., Tesli M., Melle I., Andreassen O.A., Agartz I. (2022). The Age of Violence: Mapping Brain Age in Psychosis and Psychopathy. NeuroImage Clin..

[34] Ballester P.L., Suh J.S., Ho N.C.W., Liang L., Hassel S., Strother S.C., Arnott S.R., Minuzzi L., Sassi R.B., Lam R.W. (2023). Gray Matter Volume Drives the Brain Age Gap in Schizophrenia: A SHAP Study. Schizophrenia.

[35] Xifra-Porxas A., Ghosh A., Mitsis G.D., Boudrias M.-H. (2021). Estimating Brain Age from Structural MRI and MEG Data: Insights from Dimensionality Reduction Techniques. NeuroImage.

[36] More S., Antonopoulos G., Hoffstaedter F., Caspers J., Eickhoff S.B., Patil K.R. (2023). Brain-Age Prediction: A Systematic Comparison of Machine Learning Workflows. NeuroImage.

[37] Ly M., Yu G.Z., Karim H.T., Muppidi N.R., Mizuno A., Klunk W.E., Aizenstein H.J. (2020). Improving Brain Age Prediction Models: Incorporation of Amyloid Status in Alzheimer’s Disease. Neurobiol. Aging.

[38] Han J., Kim S.Y., Lee J., Lee W.H. (2022). Brain Age Prediction: A Comparison between Machine Learning Models Using Brain Morphometric Data. Sensors.

[39] Lee W.H., Antoniades M., Schnack H.G., Kahn R.S., Frangou S. (2021). Brain Age Prediction in Schizophrenia: Does the Choice of Machine Learning Algorithm Matter?. Psychiatry Res. Neuroimaging.

[40] Kalc P., Dahnke R., Hoffstaedter F., Gaser C., Initiative A.D.N. (2024). BrainAGE: Revisited and Reframed Machine Learning Workflow. Hum. Brain Mapp..

[41] Wu F., Ma H., Guan Y., Tian L. (2023). Manifold-Based Unsupervised Metric Learning, with Applications in Individualized Predictions Based on Functional Connectivity. Biomed. Signal Process. Control.

[42] Popescu S.G., Glocker B., Sharp D.J., Cole J.H. (2021). Local Brain-Age: A U-Net Model. Front. Aging Neurosci..

[43] Borkar K., Chaturvedi A., Vinod P.K., Bapi R.S. (2022). Ayu-Characterization of Healthy Aging from Neuroimaging Data with Deep Learning and rsfMRI. Front. Comput. Neurosci..

[44] Xu L., Ma H., Guan Y., Liu J., Huang H., Zhang Y., Tian L. (2023). A Siamese Network With Node Convolution for Individualized Predictions Based on Connectivity Maps Extracted From Resting-State fMRI Data. IEEE J. Biomed. Health Inform..

[45] Valdes-Hernandez P.A., Laffitte Nodarse C., Peraza J.A., Cole J.H., Cruz-Almeida Y. (2023). Toward MR Protocol-Agnostic, Unbiased Brain Age Predicted from Clinical-Grade MRIs. Sci. Rep..

[46] Ding W., Shen X., Huang J., Ju H., Chen Y., Yin T. (2023). Brain Age Prediction Based on Resting-State Functional MRI Using Similarity Metric Convolutional Neural Network. IEEE Access.

[47] Besson P., Parrish T., Katsaggelos A.K., Bandt S.K. (2021). Geometric Deep Learning on Brain Shape Predicts Sex and Age. Comput. Med. Imaging Graph..

[48] Cheng Y., Zhang X.-D., Chen C., He L.-F., Li F.-F., Lu Z.-N., Man W.-Q., Zhao Y.-J., Chang Z.-X., Wu Y. (2023). Dynamic Evolution of Brain Structural Patterns in Liver Transplantation Recipients: A Longitudinal Study Based on 3D Convolutional Neuronal Network Model. Eur. Radiol..

[49] Ballester P.L., da Silva L.T., Marcon M., Esper N.B., Frey B.N., Buchweitz A., Meneguzzi F. (2021). Predicting Brain Age at Slice Level: Convolutional Neural Networks and Consequences for Interpretability. Front. Psychiatry.

[50] Kuchcinski G., Rumetshofer T., Zervides K.A., Lopes R., Gautherot M., Pruvo J.-P., Bengtsson A.A., Hansson O., Jönsen A., Sundgren P.C.M. (2023). MRI BrainAGE Demonstrates Increased Brain Aging in Systemic Lupus Erythematosus Patients. Front. Aging Neurosci..

[51] Gopinath K., Desrosiers C., Lombaert H. (2022). Learnable Pooling in Graph Convolutional Networks for Brain Surface Analysis. IEEE Trans. Pattern Anal. Mach. Intell..

[52] Gautherot M., Kuchcinski G., Bordier C., Sillaire A.R., Delbeuck X., Leroy M., Leclerc X., Pruvo J.-P., Pasquier F., Lopes R. (2021). Longitudinal Analysis of Brain-Predicted Age in Amnestic and Non-Amnestic Sporadic Early-Onset Alzheimer’s Disease. Front. Aging Neurosci..

[53] Hwang I., Yeon E.K., Lee J.Y., Yoo R.-E., Kang K.M., Yun T.J., Choi S.H., Sohn C.-H., Kim H., Kim J. (2021). Prediction of Brain Age from Routine T2-Weighted Spin-Echo Brain Magnetic Resonance Images with a Deep Convolutional Neural Network. Neurobiol. Aging.

[54] Chen M., Wang Y., Shi Y., Feng J., Feng R., Guan X., Xu X., Zhang Y., Jin C., Wei H. (2024). Brain Age Prediction Based on Quantitative Susceptibility Mapping Using the Segmentation Transformer. IEEE J. Biomed. Health Inform..

[55] Feng X., Lipton Z.C., Yang J., Small S.A., Provenzano F.A. (2020). Estimating Brain Age Based on a Uniform Healthy Population with Deep Learning and Structural Magnetic Resonance Imaging. Neurobiol. Aging.

[56] Bashyam V.M., Erus G., Doshi J., Habes M., Nasrallah I.M., Truelove-Hill M., Srinivasan D., Mamourian L., Pomponio R., Fan Y. (2020). MRI Signatures of Brain Age and Disease over the Lifespan Based on a Deep Brain Network and 14 468 Individuals Worldwide. Brain.

[57] Hofmann S.M., Beyer F., Lapuschkin S., Goltermann O., Loeffler M., Müller K.-R., Villringer A., Samek W., Witte A.V. (2022). Towards the Interpretability of Deep Learning Models for Multi-Modal Neuroimaging: Finding Structural Changes of the Ageing Brain. NeuroImage.

[58] Couvy-Duchesne B., Faouzi J., Martin B., Thibeau–Sutre E., Wild A., Ansart M., Durrleman S., Dormont D., Burgos N., Colliot O. (2020). Ensemble Learning of Convolutional Neural Network, Support Vector Machine, and Best Linear Unbiased Predictor for Brain Age Prediction: ARAMIS Contribution to the Predictive Analytics Competition 2019 Challenge. Front. Psychiatry.

[59] Poloni K.M., Ferrari R.J. (2022). A Deep Ensemble Hippocampal CNN Model for Brain Age Estimation Applied to Alzheimer’s Diagnosis. Expert Syst. Appl..

[60] Kianian I., Sajedi H. (2024). Brain Age Estimation with a Greedy Dual-Stream Model for Limited Datasets. Neurocomputing.

[61] Hepp T., Blum D., Armanious K., Schölkopf B., Stern D., Yang B., Gatidis S. (2021). Uncertainty Estimation and Explainability in Deep Learning-Based Age Estimation of the Human Brain: Results from the German National Cohort MRI Study. Comput. Med. Imaging Graph..

[62] Zhang Z., Jiang R., Zhang C., Williams B., Jiang Z., Li C.-T., Chazot P., Pavese N., Bouridane A., Beghdadi A. (2022). Robust Brain Age Estimation Based on sMRI via Nonlinear Age-Adaptive Ensemble Learning. IEEE Trans. Neural Syst. Rehabil. Eng..

[63] Joo Y., Namgung E., Jeong H., Kang I., Kim J., Oh S., Lyoo I.K., Yoon S., Hwang J. (2023). Brain Age Prediction Using Combined Deep Convolutional Neural Network and Multi-Layer Perceptron Algorithms. Sci. Rep..

[64] He S., Pereira D., David Perez J., Gollub R.L., Murphy S.N., Prabhu S., Pienaar R., Robertson R.L., Ellen Grant P., Ou Y. (2021). Multi-Channel Attention-Fusion Neural Network for Brain Age Estimation: Accuracy, Generality, and Interpretation with 16,705 Healthy MRIs across Lifespan. Med. Image Anal..

[65] Wood D.A., Kafiabadi S., Busaidi A.A., Guilhem E., Montvila A., Lynch J., Townend M., Agarwal S., Mazumder A., Barker G.J. (2022). Accurate Brain-age Models for Routine Clinical MRI Examinations. NeuroImage.

[66] Dular L., Pernuš F., Špiclin Ž. (2024). Extensive T1-Weighted MRI Preprocessing Improves Generalizability of Deep Brain Age Prediction Models. Comput. Biol. Med..

[67] Dular L., Špiclin Ž. (2024). BASE: Brain Age Standardized Evaluation. NeuroImage.

[68] Lim H., Joo Y., Ha E., Song Y., Yoon S., Shin T. (2024). Brain Age Prediction Using Multi-Hop Graph Attention Combined with Convolutional Neural Network. Bioengineering.

[69] Kuo C.-Y., Tai T.-M., Lee P.-L., Tseng C.-W., Chen C.-Y., Chen L.-K., Lee C.-K., Chou K.-H., See S., Lin C.-P. (2021). Improving Individual Brain Age Prediction Using an Ensemble Deep Learning Framework. Front. Psychiatry.

[70] Wang Y., Wen J., Xin J., Zhang Y., Xie H., Tang Y. (2023). 3DCNN Predicting Brain Age Using Diffusion Tensor Imaging. Med. Biol. Eng. Comput..

[71] He S., Grant P.E., Ou Y. (2022). Global-Local Transformer for Brain Age Estimation. IEEE Trans. Med. Imaging.

[72] Cheng J., Liu Z., Guan H., Wu Z., Zhu H., Jiang J., Wen W., Tao D., Liu T. (2021). Brain Age Estimation From MRI Using Cascade Networks With Ranking Loss. IEEE Trans. Med. Imaging.

[73] Leonardsen E.H., Peng H., Kaufmann T., Agartz I., Andreassen O.A., Celius E.G., Espeseth T., Harbo H.F., Høgestøl E.A., Lange A.-M.d. (2022). Deep Neural Networks Learn General and Clinically Relevant Representations of the Ageing Brain. NeuroImage.

[74] Zhang Y., Xie R., Beheshti I., Liu X., Zheng G., Wang Y., Zhang Z., Zheng W., Yao Z., Hu B. (2024). Improving Brain Age Prediction with Anatomical Feature Attention-Enhanced 3D-CNN. Comput. Biol. Med..

[75] Bellantuono L., Marzano L., La Rocca M., Duncan D., Lombardi A., Maggipinto T., Monaco A., Tangaro S., Amoroso N., Bellotti R. (2021). Predicting Brain Age with Complex Networks: From Adolescence to Adulthood. NeuroImage.

[76] Peng H., Gong W., Beckmann C.F., Vedaldi A., Smith S.M. (2021). Accurate Brain Age Prediction with Lightweight Deep Neural Networks. Med. Image Anal..

[77] Armanious K., Abdulatif S., Shi W., Salian S., Küstner T., Weiskopf D., Hepp T., Gatidis S., Yang B. (2021). Age-Net: An MRI-Based Iterative Framework for Brain Biological Age Estimation. IEEE Trans. Med. Imaging.

[78] Fu Y., Huang Y., Zhang Z., Dong S., Xue L., Niu M., Li Y., Shi Z., Wang Y., Zhang H. (2023). OTFPF: Optimal Transport Based Feature Pyramid Fusion Network for Brain Age Estimation. Inf. Fusion.

[79] Simonyan K., Zisserman A. Very Deep Convolutional Networks for Large-Scale Image Recognition. https://arxiv.org/abs/1409.1556v6.

[80] He K., Zhang X., Ren S., Sun J. Deep Residual Learning for Image Recognition. https://arxiv.org/abs/1512.03385v1.

[81] Szegedy C., Liu W., Jia Y., Sermanet P., Reed S., Anguelov D., Erhan D., Vanhoucke V., Rabinovich A. Going Deeper with Convolutions. https://arxiv.org/abs/1409.4842v1.

[82] Chollet F. Xception: Deep Learning With Depthwise Separable Convolutions. Proceedings of the IEEE Conference on Computer Vision and Pattern Recognition.

[83] Huang G., Liu Z., van der Maaten L., Weinberger K.Q. Densely Connected Convolutional Networks. https://arxiv.org/abs/1608.06993v5.

[84] Tan M., Le Q.V. EfficientNet: Rethinking Model Scaling for Convolutional Neural Networks. https://arxiv.org/abs/1905.11946v5.

[85] Ronneberger O., Fischer P., Brox T. U-Net: Convolutional Networks for Biomedical Image Segmentation. https://arxiv.org/abs/1505.04597v1.

[86] Scarselli F., Gori M., Tsoi A.C., Hagenbuchner M., Monfardini G. (2009). The Graph Neural Network Model. IEEE Trans. Neural Netw..

[87] Lin L., Xiong M., Zhang G., Kang W., Sun S., Wu S. (2023). Initiative Alzheimer’s Disease Neuroimaging A Convolutional Neural Network and Graph Convolutional Network Based Framework for AD Classification. Sensors.

[88] Minar M.R., Naher J. Recent Advances in Deep Learning: An Overview. https://arxiv.org/abs/1807.08169v1.

[89] Alzheimer’s Disease Neuroimaging Initiative (ADNI). https://www.neurology.org/doi/10.1212/WNL.0b013e3181cb3e25.

[90] Sudlow C., Gallacher J., Allen N., Beral V., Burton P., Danesh J., Downey P., Elliott P., Green J., Landray M. (2015). UK Biobank: An Open Access Resource for Identifying the Causes of a Wide Range of Complex Diseases of Middle and Old Age. PLoS Med..

[91] Thompson P.M., Jahanshad N., Ching C.R.K., Salminen L.E., Thomopoulos S.I., Bright J., Baune B.T., Bertolín S., Bralten J., Bruin W.B. (2020). ENIGMA and Global Neuroscience: A Decade of Large-Scale Studies of the Brain in Health and Disease across More than 40 Countries. Transl. Psychiatry.

